# Bioactivity-Guided Identification of Ethyl Gallate from Pecan (*Carya illinoinensis*) and Its Protective Effects Against Oxidative Stress and Amyloid-β-Induced Cognitive Impairment

**DOI:** 10.3390/foods15183316

**Published:** 2026-09-19

**Authors:** Chan Kyu Park, Dong-Hoon Shin

**Affiliations:** Department of Food and Biotechnology, Korea University, Sejong 30019, Republic of Korea; dhshin@korea.ac.kr

**Keywords:** amyloid β, bioactive compound, cognitive function, ethyl gallate, oxidative stress, pecan

## Abstract

Pecan (*Carya illinoinensis*) is an edible nut rich in phytochemicals with potential health-promoting properties; however, the bioactive constituents responsible for its neuroprotective effects remain insufficiently characterized. This study aimed to identify bioactive constituents from pecan through bioactivity-guided fractionation and to evaluate the protective effects of pecan extract and the identified compound against oxidative stress and amyloid beta (Aβ)_1–42_-induced cognitive impairment. Pecan ethanolic extract exhibited antioxidant and cytoprotective activities in PC12 cells exposed to hydrogen peroxide-induced oxidative stress. Sequential liquid–liquid fractionation, silica gel open-column chromatography, and preparative thin-layer chromatography, coupled with repeated bioactivity screening, progressively tracked the active fractions and led to the identification of ethyl gallate as a bioactive constituent of pecan extract by high-performance liquid chromatography and gas chromatography–mass spectrometry. The biological relevance of these findings was further evaluated in Aβ_1–42_-injected male mice. Dietary administration of pecan extract significantly improved spontaneous alternation performance in the Y-maze test, whereas ethyl gallate improved Y-maze performance and memory retention in the passive avoidance test, without apparent systemic toxicity under the experimental conditions. Although brain malondialdehyde levels showed a numerical decrease following ethyl gallate administration, the differences were not statistically significant. Collectively, these findings demonstrate the bioactivity-guided linkage of ethyl gallate to an active pecan fraction and provide preclinical evidence supporting further investigation of pecan-derived bioactives as food-derived functional ingredients relevant to cognitive health.

## 1. Introduction

Over recent decades, the global prevalence of dementia—most notably Alzheimer’s disease (AD)—has surged, emerging as a critical public health challenge due to its profound economic and societal impacts. A central focus of Alzheimer’s research has been the pathological buildup of amyloid beta (Aβ) peptides in the brain, widely regarded as a key driver of neurodegeneration and memory loss in AD patients [1]. However, the credibility of this “amyloid cascade hypothesis” has come under scrutiny following allegations of scientific misconduct. A notable controversy erupted in 2022, when investigative reports exposed possible image manipulation in influential research implicating a particular Aβ oligomer, Aβ*56, in cognitive dysfunction [2]. This incident sparked intense debate about the reliability of foundational Aβ studies and cast doubt on the robustness of the amyloid-based disease model. Nevertheless, the amyloid hypothesis remains a central framework in AD research, as considerable evidence still supports Aβ’s role in disease progression. Current studies increasingly recognize that AD is a multifactorial disorder and thus explore the broader network of pathogenic mechanisms—including tau pathology, neuroinflammation, oxidative stress, and synaptic dysfunction—to achieve a more integrated understanding of the disease [3,4].

Although several AD therapies have gained US Food and Drug Administration approval in recent years, monoclonal antibodies such as aducanumab, lecanemab, and donanemab have expanded the therapeutic options for AD. Nevertheless, currently available therapies exert only modest effects in slowing cognitive decline and fail to provide a definitive cure or preventive protection [5]. Interventions involving bioactive compounds from plant-based foods—such as flavonoids, polyphenols, and unsaturated fatty acids—are gaining prominence due to their potential neuroprotective effects. For instance, Yang et al. demonstrated that a dietary pigment, curcumin, inhibited the formation of Aβ oligomers (even more potent than naproxen) and thus prevented Aβ fibril formation in vivo [6]. Similarly, it was reported that a 6-month intervention of extra-virgin olive oil ameliorated cognition and neuropathology markers (e.g., peptide levels and deposition) in vivo [7]. These natural compounds may act through antioxidant, anti-inflammatory, and synaptic support mechanisms, offering a promising avenue for delaying or even preventing AD onset.

Among various nutrient-rich plant foods under investigation, pecans (*Carya illinoinensis*) have emerged as a promising candidate due to their high content of unsaturated fatty acids, phenolic compounds, tocopherols, and flavonoids—all of which have been linked to neuroprotective effects. Pecans are particularly rich in ellagic acid, catechins, and γ-tocopherol, which exhibit strong antioxidant and anti-inflammatory activity, potentially mitigating oxidative stress and neuroinflammation—two key contributors to AD pathogenesis [8]. In a recent clinical study, daily pecan consumption was associated with increased plasma antioxidant capacity and reduced lipid peroxidation markers in healthy adults, indicating potential systemic benefits relevant to brain health [9]. Additionally, preclinical evidence indicates that polyphenols derived from nuts possess the ability to mitigate Aβ-induced neurotoxicity and support synaptic integrity in models of neurodegeneration [10]. These bioactives may modulate oxidative and inflammatory pathways in the brain, thereby reducing neuronal damage and preserving cognitive function in AD models. These findings collectively indicate that pecan-enriched diets may offer a multifaceted dietary approach to prevent or delay the onset of AD.

Within this context, pecan, a nut rich in phenolic compounds, unsaturated fatty acids, and dietary fiber, has emerged as a promising functional food due to its potent antioxidant and anti-inflammatory properties. Animal and cellular studies have demonstrated that polyphenols derived from pecans can reduce systemic inflammation and oxidative stress, which are key contributors to neurodegeneration and cognitive decline [11]. However, direct evidence of pecan compounds modifying Aβ pathology in AD remains sparse, although the anti-inflammatory and antioxidative effects observed in peripheral models provide a compelling rationale for future preclinical dementia-focused investigations. Consequently, the present study aims to explore whether pecan can mitigate Aβ-induced neurotoxicity and identify specific compound(s) active against this core feature of AD. To this end, the ethanolic extract of pecan was subjected to multiple separation and analytical techniques, in parallel with both in vitro and in vivo efficacy assessments.

## 2. Materials and Methods

### 2.1. Materials

The following reagents were used in this study: 2′,7′-dichlorofluorescin diacetate (DCF-DA), 3-(4,5-dimethylthiazol-2-yl)-2,5-diphenyl tetrazolium bromide (MTT), dimethyl sulfoxide (DMSO), hydrogen peroxide, and L-ascorbic acid (vitamin C), all obtained from Sigma-Aldrich (St. Louis, MO, USA). Silica gel was procured from Merck (Darmstadt, Germany), while amyloid beta peptide was purchased from Bachem Holding (Bubendorf, Switzerland). Serum transaminase assay kits were provided by the Asan Pharmaceutical (Seoul, Republic of Korea). All other reagents used were of analytical grade, unless stated otherwise.

### 2.2. Cell Culture Conditions

Rat pheochromocytoma cell line 12 (PC12) (CRL-1721, ATCC; Manassas, VA, USA) was maintained as we recently described [12]. In brief, the cells were cultured in RPMI 1640 medium supplemented with 10% heat-inactivated horse serum, 5% fetal bovine serum, and 1% antibiotic–antimycotic solution (Gibco-Invitrogen; Grand Island, NY, USA). The cells were incubated at 37 °C in a humidified atmosphere containing 5% CO_2_ and were subcultured upon reaching 80–90% confluency. Culture media were replaced at least three times per week. Sodium bicarbonate (Sigma-Aldrich), phosphate buffers (Junsei Chemical, Tokyo, Japan), and potassium chloride (Showa Chemicals, Tokyo, Japan) were used as supporting reagents. Experimental procedures followed previously described protocols [12].

### 2.3. Measurement of Intracellular Reactive Oxygen Species (ROS) Levels

Intracellular ROS levels were measured using the 2′,7′-dichlorofluorescein diacetate (DCF-DA) assay, following protocols described in our previous studies [13,14]. PC12 cells were seeded into 96-well plates at a density of 1.0 × 10^5^ to 5.0 × 10^5^ cells/mL (100 μL per well). After a 48 h pretreatment with each sample extract (1 mg/mL), cells were exposed to freshly prepared hydrogen peroxide (100 μM) or vehicle control for 2 h. Subsequently, 250 μM DCF-DA was added, and the cells were incubated for 50 min. Fluorescence intensity, reflecting intracellular ROS generation, was detected using a GENios microplate fluorometer (TECAN; Männedorf, Switzerland) at 485 nm excitation and 535 nm emission wavelengths.

### 2.4. Measurement of Cytotoxicity

The cytotoxicity of the test samples was evaluated using the standard MTT reduction assay, as previously described [14]. Briefly, PC12 cells were seeded in 96-well plates at a density of 1.0 × 10^5^ to 5.0 × 10^5^ cells/mL (100 μL per well) and allowed to adhere overnight. The cells were then pretreated with test extracts (1 mg/mL) for 48 h, followed by a 2 h exposure to 200 μM hydrogen peroxide to induce oxidative stress. After treatment, 0.25 mg/mL MTT solution was added to each well, and the plates were incubated for 3 h at 37 °C in a humidified incubator with 5% CO_2_. During this period, metabolically active cells reduced MTT to insoluble formazan crystals. The culture medium was then discarded, and 150 μL of DMSO was added to dissolve the formazan. Absorbance was measured at 570 nm with a reference wavelength of 630 nm using a GENios microplate reader (TECAN). Cell viability was expressed as a percentage relative to untreated control cells.

### 2.5. Preparation of Plant Extracts

Seventeen dried edible and medicinal plant materials, comprising commonly consumed food plants and traditionally used medicinal plants, were purchased from Kyungdong Market (Seoul, Republic of Korea). Prior to extraction, the botanical identity of each material was confirmed based on morphological characteristics by an expert from the Department of GreenBio Science, Gyeongsang National University. The same identification procedure was consistently applied to all samples. The scientific names and details of the plant materials are provided in Table 1. The samples were stored at 4 °C until extraction. Approximately 50 g of each dried sample was ground into a fine powder and extracted with 80% ethanol at a sample-to-solvent ratio of 1:5 (*w*/*v*) by stirring at room temperature for 24 h. The mixture was filtered through Whatman No. 42 filter paper (Cytiva, Marlborough, MA, USA), and the remaining plant residue was subjected to two additional extractions under identical conditions. The combined filtrates were concentrated under reduced pressure at 40 °C using a rotary evaporator (N-1100, EYELA, Tokyo Rikakikai Co., Ltd., Tokyo, Japan), and the resulting dried extracts were stored at −20 °C until use. For the screening assays, each extract was dissolved in 5% DMSO to a final concentration of 1 mg/mL.

The initial comparison of the 17 extracts was conducted as a preliminary screening to prioritize candidates for subsequent bioactivity-guided fractionation. For each extract, DCF-DA and MTT measurements were obtained from four technical replicate wells within a single screening experiment, and the results are presented as mean ± SD in Table 1. No inferential statistical comparisons among the 17 extracts were performed at this screening stage.

### 2.6. Liquid–Liquid Fractionation

Approximately 6 kg of pecan kernels were extracted with 80% ethanol (1:5, *w*/*v*) by stirring at room temperature for 24 h. To maximize extraction efficiency, the procedure was repeated, and the combined filtrates were concentrated under reduced pressure to yield 814 g of crude ethanolic extract. A 63 g portion of this extract was reserved for in vivo studies. The remaining extract was dissolved in 750 mL of distilled water to initiate sequential liquid–liquid partitioning. For the first step, three successive volumes of n-hexane (2250 mL each) were added, thoroughly mixed, and allowed to stand for 24 h to ensure complete phase separation. The resulting n-hexane fractions were collected and pooled. The aqueous layer was then extracted similarly with chloroform (3 × 2250 mL), followed by ethyl acetate (3 × 2250 mL), using the same procedure. Each organic layer was separately concentrated under reduced pressure at 40 °C. The dried fractions (n-hexane, chloroform, and ethyl acetate) were stored and later screened for biological activity using MTT and DCF-DA assays to identify the most active component.

### 2.7. Silica Gel Open Column Chromatography

Following liquid–liquid fractionation, the most bioactive fraction—identified via MTT and DCF-DA assays—was subjected to further purification using open-column chromatography on silica gel. Silica gel (525 g) was activated by heating at 100 °C for at least 2 h and then uniformly packed into a glass column with gentle tapping to eliminate air gaps. The selected fraction was dissolved in a minimal volume of the initial mobile phase and carefully loaded onto the top of the silica bed. Elution was carried out using a stepwise gradient system of chloroform and ethanol, starting from 100:0 (*v*/*v*) and gradually increasing the ethanol content (e.g., 90:10, 80:20, 70:30) up to 0:100. The eluent was passed through the column under gravity, and fractions were collected sequentially in 1260 mL volumes. A total of 33 fractions were obtained, concentrated by evaporation, and subsequently evaluated for cytoprotective and antioxidant activity using MTT and DCF-DA assays.

### 2.8. Preparative Thin Layer Chromatography (TLC)

Preparative TLC was employed to isolate the active compound from the fraction exhibiting the most potent antioxidant activity in prior assays. The selected fraction was dissolved in absolute ethanol at a concentration of 250 mg/mL. A total volume of 40 µL of the sample solution, corresponding to 10 mg of the fraction, was applied to the same spot on a 30 × 30 cm silica gel TLC plate (Merck) in two successive applications of 20 µL each. The plate was developed using chloroform/ethanol (60:40, *v*/*v*) as the mobile phase. After development, the separated bands were visualized under ultraviolet light at 254 and 365 nm. Individual bands were carefully scraped from the plate, eluted with ethanol, and concentrated. The resulting subfractions were evaluated for cytoprotective and antioxidant effects using MTT and DCF-DA assays.

### 2.9. High-Performance Liquid Chromatography (HPLC) Analysis

HPLC analysis was performed using a YL9160 photodiode array detector (YL Instruments Co., Ltd., Anyang, Republic of Korea) equipped with an Aegispak C18-L column (4.6 × 250 mm, 5 µm, 100 Å; YoungJin Biochrom, Seongnam, Republic of Korea). The column temperature was maintained at 23 °C. Chromatographic separation was performed under isocratic conditions using methanol/acetonitrile/10 mM ammonium acetate containing 0.1% formic acid (10:25:65, *v*/*v*/*v*) as the mobile phase at a flow rate of 0.5 mL/min for 30 min. The sample was dissolved in ethanol at a concentration of 60 mg/mL, and 20 µL was injected for analysis. An authentic ethyl gallate standard was prepared at 1 mg/mL for comparison.

### 2.10. Gas Chromatography–Mass Spectrometry (GC-MS) Analysis

GC–MS analysis was conducted using an Agilent 6890 Plus gas chromatograph coupled to a 5977 N quadrupole mass selective detector (Agilent Technologies; Palo Alto, CA, USA). Separation was achieved on a J&W Scientific capillary column (30 m × 0.25 mm i.d., 0.25 μm film thickness) coated with a 5% diphenyl/95% dimethylsiloxane stationary phase. No chemical derivatization, including silylation, was performed prior to GC–MS analysis. The system was operated in electron impact (EI) ionization mode at an electron energy of 79 eV, and spectra were acquired in full-scan mode over an *m/z* range of 50–700. The obtained mass spectrum was compared with the Wiley 7N mass spectral library, and the peak assigned to ethyl gallate showed a high spectral library match quality (Qual = 98). Compound identification was further supported by HPLC analysis, in which the corresponding chromatographic peak showed co-elution with an authentic ethyl gallate standard.

### 2.11. In Vivo Experiment I and II: Mice Intervention Studies Using Pecan Crude Extract and Its Active Compound, Ethyl Gallate

Five-week-old male ICR (Institute of Cancer Research) mice were obtained from Daehan Biolink (Chungnam, Republic of Korea) and randomly assigned to experimental groups (*n =* 8 per group). Each experimental group was housed in a separate cage under controlled conditions (12 h light/dark cycle, 55% humidity, 23–25 °C) with ad libitum access to food and water. For crude extract treatment (In vivo experiment I), Pecan extract was incorporated into the chow diet at concentrations calculated based on the initial body weight of the animals and the expected average daily food intake to provide target doses of 400, 800, and 1200 mg/kg body weight/day. The experimental diets were provided ad libitum to minimize the potential stress associated with repeated oral administration. For ethyl gallate treatment (In vivo experiment II), ethyl gallate was similarly incorporated into the chow diet at concentrations calculated to provide target doses of 10, 20, and 40 mg/kg body weight/day based on body weight and expected average daily food intake. These doses were selected independently for the in vivo evaluation of ethyl gallate and were not derived from, or intended to be dose-equivalent to, the ethyl gallate content of the pecan extract administered in In vivo experiment I. The prepared experimental diets were divided into individual portions, protected from light, and stored at −20 °C until use. The remaining diet in the feeders was replaced with freshly prepared experimental diet every other day. Food consumption was not quantitatively monitored during the intervention period; therefore, individual daily intake of the test materials could not be precisely determined. After three weeks of dietary intervention, mice in the Aβ-treated groups received a single intracerebroventricular (ICV) injection of Aβ_1–42_ peptide (410 pmol/mouse), whereas mice in the normal control group received an equivalent dose of the reverse-sequence peptide Aβ_42–1_. Both peptide solutions were freshly prepared on the day of administration by dissolving the respective peptides in 0.85% sodium chloride (*w*/*v*). No additional aggregation, aging, or pre-incubation step was performed, and the freshly prepared peptide solutions were used immediately for ICV administration. Each peptide solution was administered at a volume of 5 μL/mouse. ICV injection was performed according to a previously described procedure [15], with minor modification. The injection site was located approximately 1.0 mm posterior to bregma and 1.8 mm lateral to the sagittal suture. A microsyringe fitted with a 26-gauge needle was inserted perpendicular to the skull surface to a depth of 2.5 mm, and 5 μL of peptide solution was administered per mouse.

### 2.12. Y-Maze Test

Spontaneous alternation behavior in the Y-maze was used to assess immediate working memory five days following Aβ administration, as previously described [12]. Briefly, the Y-maze apparatus, constructed from black-painted plastic, comprised three arms (33 cm long × 10 cm wide × 15 cm high) arranged at 120° angles. Each mouse was placed at the end of one arm and allowed to explore the maze freely for 8 min. An arm entry was recorded only when the mouse’s hind limbs were fully inside an arm. Alternation behavior was defined as sequential entries into all three arms in overlapping triplet sets. The percentage of spontaneous alternation was calculated using the formula: [Number of actual alternations/(Total arm entries − 2)] × 100.

### 2.13. Passive Avoidance Test

The passive avoidance test was conducted to evaluate aversive learning and memory, following previously established protocols [12]. In brief, the passive avoidance test apparatus consisted of two compartments: one illuminated and one dark. During the training phase, each mouse was placed in the lighted compartment, and upon entering the dark compartment, received a mild foot shock (0.5 mA for one second). Twenty-four hours later, a retention trial was performed in which the mouse was reintroduced into the illuminated compartment, and the latency to re-enter the dark compartment (step-through latency) was recorded. A maximum latency cutoff of 300 s was imposed.

### 2.14. Evaluation of Acute Liver Toxicity

After behavior tests, the acute toxicity of samples was measured using the serum transaminase reagents kit (AM 101-K, Asan Pharmaceutical) as instructed by the manufacturer’s manuals.

### 2.15. Assessment of Lipid Peroxidation Using the ICR Mice Brain

The lipid peroxidation level in brain tissue was determined using the thiobarbituric acid-reactive substances (TBARS) assay by measuring malondialdehyde (MDA), as described previously [12]. Briefly, 80 μL of brain tissue homogenate was mixed with 480 μL of 1% H_3_PO_4_ and 160 μL of 0.67% (*w*/*v*) thiobarbituric acid (TBA) solution. The reaction mixture was heated at 95 °C for 45 min and subsequently cooled for 10 min. After cooling, 640 μL of n-butanol was added, and the mixture was centrifuged at 6000 rpm for 3 min. A 500 μL aliquot of the resulting supernatant was mixed with 500 μL of n-butanol, and the absorbance was measured at 532 nm. MDA levels were quantified as MDA equivalents using 1,1,3,3-tetramethoxypropane as the standard.

### 2.16. Animal Ethics Statement

All animal experiments were conducted in accordance with the Guide for the Care and Use of Laboratory Animals and the ARRIVE guidelines. The protocol was approved by the Institutional Animal Care and Use Committee of Korea University (approval code: KUIACUC-2017-142; approval date: 27 October 2017).

### 2.17. Statistical Analysis

In the study, all data are expressed as mean ± standard deviation. Normality of distribution was evaluated using the Shapiro–Wilk test. For non-normally distributed data, the Kruskal–Wallis test was employed, followed by Dunn’s multiple comparison test. If data met the assumptions of normality and homogeneity of variance, one-way ANOVA was performed, followed by Fisher’s least significant difference post hoc test. Statistical significance was defined as *p* < 0.05. All analyses were conducted using GraphPad Prism version 8.0.2 (GraphPad Software; San Diego, CA, USA).

## 3. Results and Discussion

### 3.1. Screening for Protective Effect of Plant Extracts Using a PC12 Cell Model

To identify extracts with potent antioxidant activity, a total of 17 edible plant ethanolic extracts were screened using DCF-DA and MTT assays under hydrogen peroxide-induced oxidative stress in PC12 cells. This model was selected based on the well-established link between Aβ accumulation and elevated ROS production in the brains of AD patients, which contributes to neuronal dysfunction and cognitive decline [16]. Among the tested samples, pecan (*C. illinoinensis*) extract showed comparatively high protective activity in both screening assays, with a protective effect of 78.5% in the DCF-DA assay and cell viability of 78.5% in the MTT assay (Table 1). Based on this screening profile, pecan extract was prioritized for subsequent bioactivity-guided fractionation to identify candidate bioactive constituents.

### 3.2. Bioactivity-Guided Fractionation and Isolation Identified Ethyl Gallate as an Active Compound of Pecan Extract

**Figure 1:**
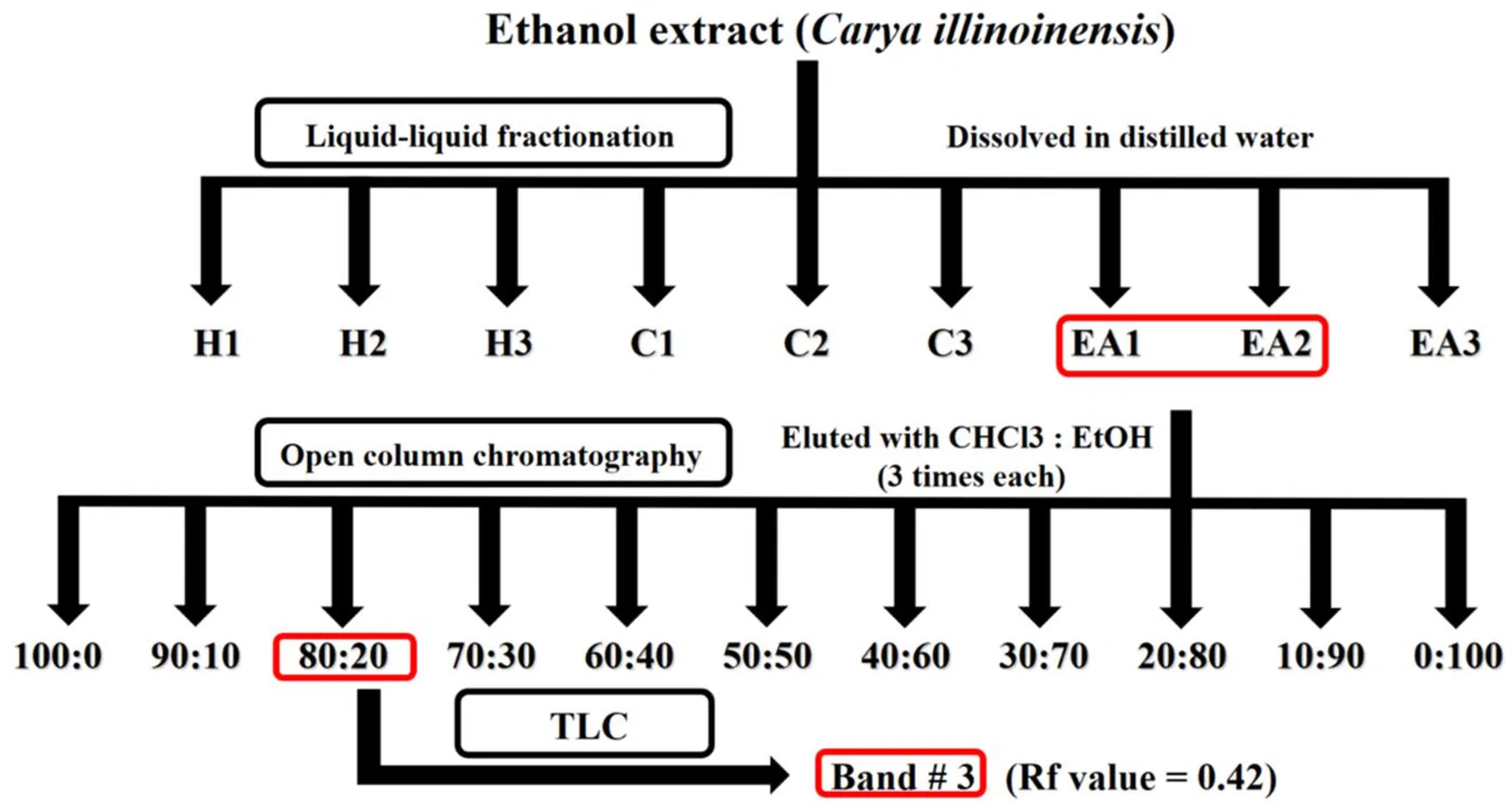
Schematic summary of isolation of protective component from *Carya illinoinensis* extract. Red squares indicate the fractions and band selected for subsequent fractionation or analysis based on their bioactivity.

**Figure 2:**
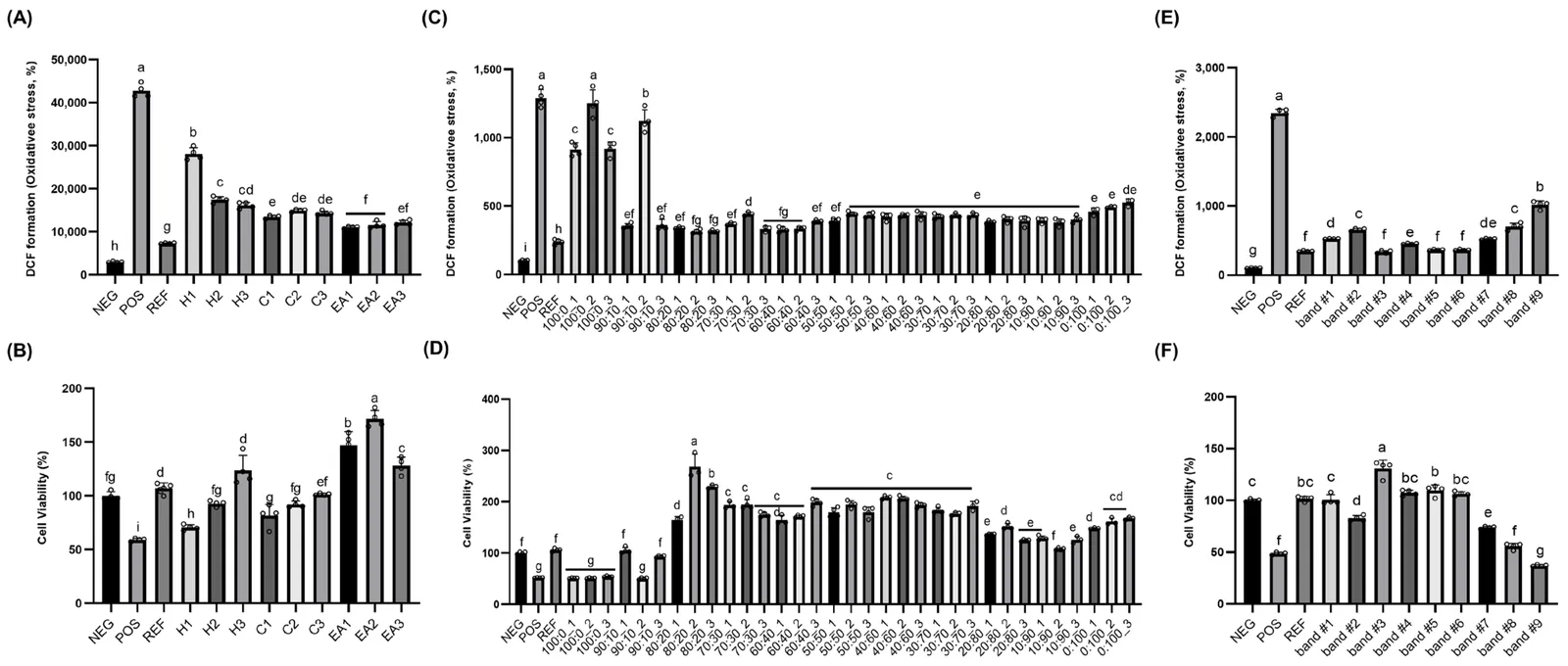
Protective effects of *Carya illinoinensis* extract fractions against hydrogen peroxide-induced oxidative stress and cytotoxicity in PC12 cells. (**A**,**C**,**E**) Intracellular ROS levels were measured using the DCF-DA assay following treatment with fractions obtained from (**A**) liquid–liquid partitioning (H1–H3, C1–C3, and EA1–EA3), (**C**) silica gel open-column chromatography (eluted with CHCl_3_:EtOH gradients), and (**E**) preparative thin-layer chromatography (TLC bands 1–9), followed by exposure to 100 μM H_2_O_2_. (**B**,**D**,**F**) Corresponding cell viability was assessed using the MTT assay after treatment with the same respective fractions, followed by exposure to 200 μM H_2_O_2_. Vitamin C (REF, 100 μM) was used as a reference antioxidant. Untreated (NEG) and H_2_O_2_-treated (POS) groups were included as controls. Data are presented as mean ± SD of four technical replicate wells within a single experiment (*n =* 4). Statistical significance was assessed using the Kruskal–Wallis test followed by Dunn’s multiple comparisons test. Different lowercase letters indicate statistically significant differences among groups (*p* < 0.05), whereas groups sharing the same letter are not significantly different. Abbreviations: NEG, untreated group; POS, hydrogen peroxide treated group; REF, vitamin C.

To identify the active constituents responsible for the protective effects of pecan extract, a multi-step separation approach was employed (Figure 1). The crude ethanolic extract was first subjected to liquid–liquid partitioning using n-hexane, chloroform, and ethyl acetate. Each solvent extraction was repeated three times to maximize yield, and the resulting organic layers were concentrated under reduced pressure at 40 °C. All fractions were then evaluated for antioxidant and cytoprotective activities using DCF-DA and MTT assays in PC12 cells. In the DCF-DA assay, the positive control group (POS; 100 μM hydrogen peroxide) exhibited approximately 15-fold higher intracellular ROS levels compared to the untreated negative control (NEG), confirming strong oxidative stress induction [Figure 2A]. Treatment with the reference antioxidant (REF; 100 μM vitamin C) significantly reduced ROS levels, validating the assay system. While all pecan-derived fractions demonstrated some degree of ROS inhibition, ethyl acetate (EA1–EA3) groups were superior in terms of ROS suppression. In the MTT assay, on the other hand, exposure to hydrogen peroxide (POS) reduced cell viability to nearly 40% of NEG, indicating marked cytotoxicity. REF treatment restored viability above baseline levels. Among the fractions, ethyl acetate (EA1–EA2) showed meaningful protective effects [Figure 2B]. Based on these results, the EA1 and EA2 fractions were selected for further purification and compound identification.

The EA1 and EA2 fractions, which showed potent protective effects, were pooled and further purified using open-column chromatography on silica gel. A stepwise elution was performed using chloroform–ethanol solvent gradients, and three subfractions were collected per gradient condition, yielding a total of 33 fractions. All subfractions were evaluated for antioxidant and cytoprotective activity in PC12 cells using DCF-DA and MTT assays. DCF-DA assay results revealed that fractions [i.e., 80:20_1 through 80:20_3 fractions in Figure 2C] eluted with 80:20 chloroform–ethanol mixture exhibited marked suppression of intracellular ROS levels [Figure 2C]. In the MTT assay, similarly, the second fraction [i.e., 80:20_2 in Figure 2D] obtained with 80:20 chloroform–ethanol displayed the highest enhancement in cell viability. Consequently, all 80:20 eluted subfractions were combined and concentrated for the TLC separation in which a 60:40 chloroform–ethanol solvent system. After development, bands were visualized under UV light at 254 and 365 nm. A total of nine distinct bands were scraped, eluted with ethanol, and assessed for bioactivity. Among these, Band #3 (Rf = 0.42) exhibited the strongest ROS inhibition and the greatest increase in cell viability, outperforming other subfractions in both assays [Figure 2E,F].

**Figure 3:**
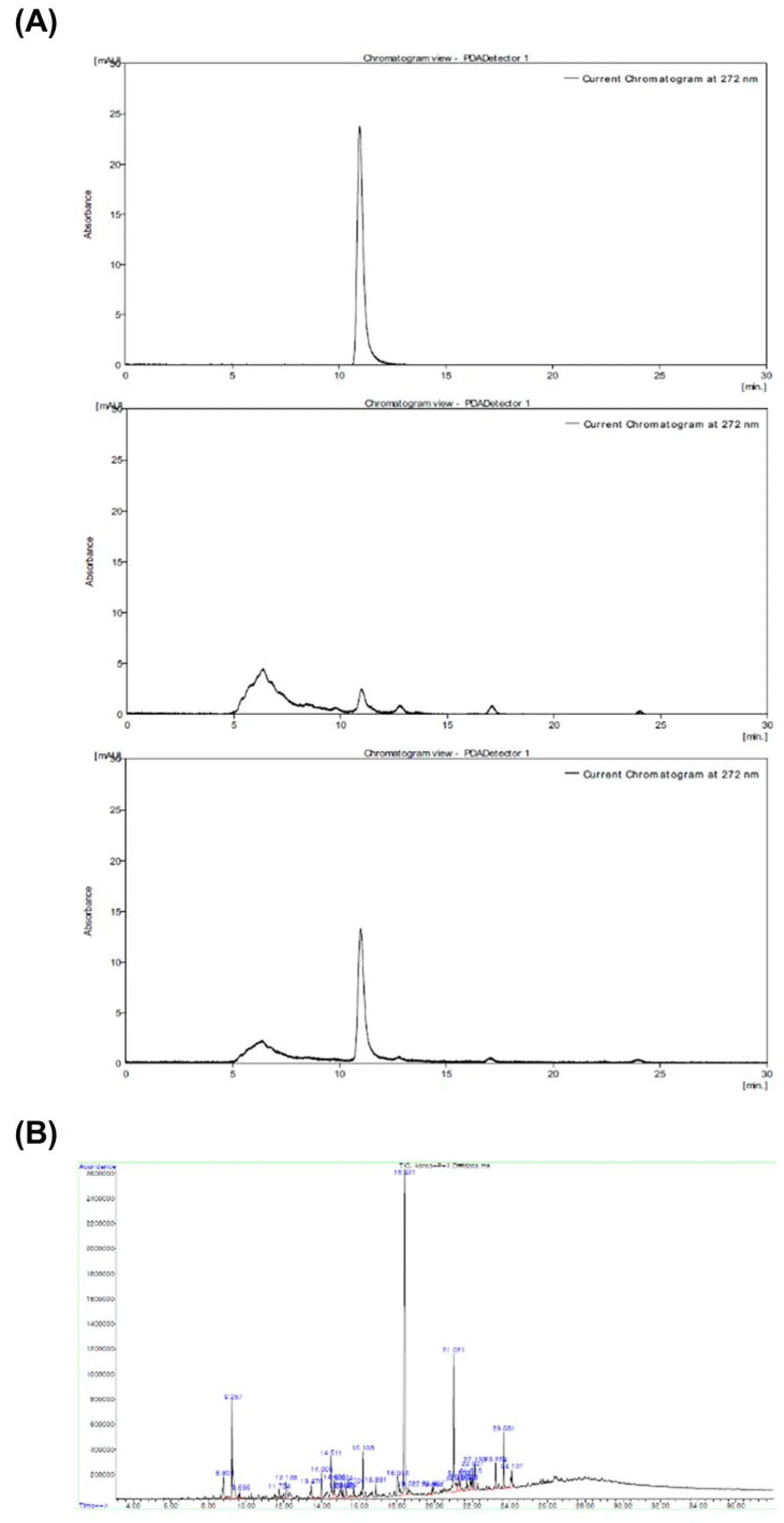
Identification of ethyl gallate isolated from *Carya illinoinensis* extract using HPLC and GC–MS analyses (**A**) HPLC analysis was conducted using an Aegispak C18-L column (5 μm, 100 Å, 4.6 × 250 mm) equipped with a YL9160 photodiode array detector. The mobile phase consisted of methanol–acetonitrile–10 mM ammonium acetate containing 0.1% formic acid (10:25:65, *v*/*v*/*v*), delivered at a flow rate of 0.5 mL/min. The detection wavelength was set at 272 nm, and the injection volume was 20 μL. HPLC chromatograms recorded at 272 nm show a distinct peak at approximately 12 min in the ethyl gallate standard (top), *CI.* extract (middle), and their co-injection mixture (bottom), confirming peak identity by co-elution. (**B**) GC–MS analysis was performed using an Agilent 6890 Plus GC system equipped with a 5977 N mass selective detector and a DB-5MS column (30 m × 0.25 mm, 0.25 μm). The sample was analyzed in electron impact mode (79 eV), and spectra were recorded in full scan mode (*m*/*z* 50–700). A major peak was observed at 18.421 min, and the corresponding EI mass spectrum showed a high match with the ethyl gallate spectrum in the Wiley 7N library. Together with HPLC co-elution using an authentic standard, these results supported the identification of the isolated compound as ethyl gallate.

The selected TLC band was extracted with ethanol, evaporated under reduced pressure at 40 °C, and re-dissolved for HPLC analysis; a prominent peak was observed at a retention time of approximately 12 min [Figure 3A]. Subsequent GC–MS analysis revealed a major peak at approximately 18.421 min [Figure 3B], and the corresponding EI mass spectrum was consistent with the ethyl gallate reference spectrum in the Wiley 7N library. Compound identification was further supported by HPLC co-elution with an authentic ethyl gallate standard. Spiking the isolated fraction with the standard resulted in amplification of the corresponding chromatographic peak without the appearance of an additional peak [Figure 3A]. Taken together, the HPLC co-elution and GC–MS spectral matching supported the identification of ethyl gallate as a bioactive constituent of the isolated fraction.

### 3.3. Pecan Extract Improved Aβ_1–42_-Induced Behavioral Deficits In Vivo

**Figure 4:**
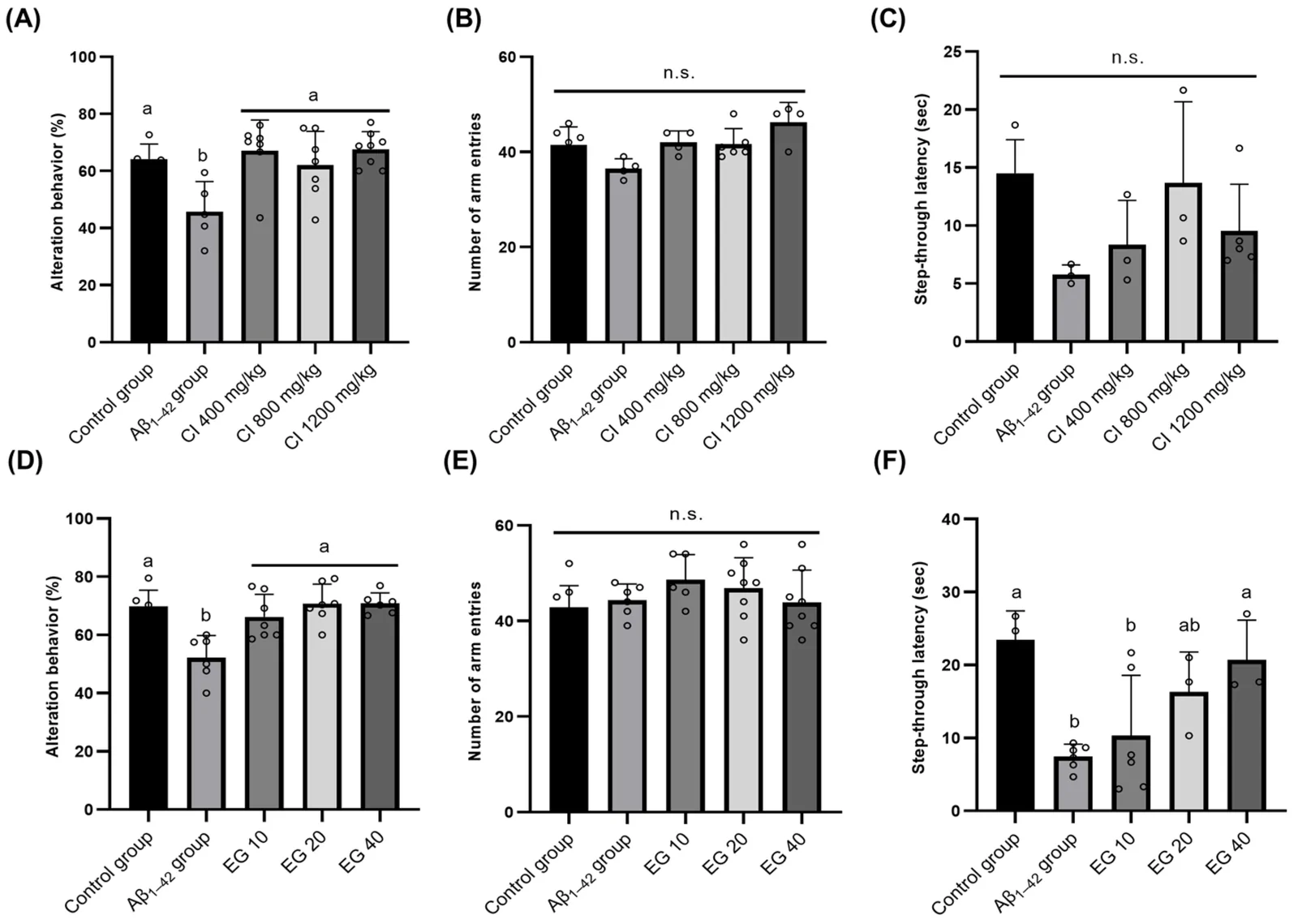
Effects of *Carya illinoinensis* extract and ethyl gallate on cognitive performance in a mouse model of Aβ_1–42_-induced cognitive impairment. Effects of *Carya illinoinensis* extract (CI) and ethyl gallate (EG) supplementation on (**A**,**D**) Y-maze test, (**B**,**E**) total arm entries, and (**C**,**F**) passive avoidance test. Control groups were injected with Aβ_42–1_, a reverse-sequence peptide of Aβ_1–42_ used as a non-toxic control. The Aβ_1–42_ groups were injected with 410 pmol of Aβ_1–42_ per mouse. Treatment groups received dietary supplementation with CI (400, 800, and 1200 mg/kg) or EG (10, 20, and 40 mg/kg) mixed in the chow for three weeks before Aβ_1–42_ injection. Data are presented as mean ± SD (*n =* 8 individual mice per group). Statistical significance was assessed using the Kruskal–Wallis test followed by Dunn’s multiple comparisons test. Different lowercase letters indicate statistically significant differences among groups (*p* < 0.05), whereas groups sharing the same letter are not significantly different. Abbreviations: *CI*, *Carya illinoinensis* extract; EG, ethyl gallate; Aβ, amyloid beta; n.s., not significant.

An in vivo intervention study was conducted to evaluate the neuroprotective efficacy of pecan extract in mice subjected to Aβ_1–42_-induced cognitive deficits. After the intervention, no significant differences were observed in body or brain weight among the groups. Similarly, no significant differences were observed in serum AST or ALT levels. Working memory performance was assessed via the Y-maze test, where the percentage of spontaneous alternation served as the primary outcome measure. Aβ-injected mice [i.e., Aβ_1–42_ group in Figure 4A] showed significantly reduced alternation behavior compared to the control group (*p* < 0.05), confirming Aβ-induced working memory impairment. Oral administration of pecan extract at doses of 400, 800, and 1200 mg/kg significantly improved alternation performance relative to the Aβ_1–42_ group, indicating a protective effect; however, no clear dose-dependent trend was observed, as all three treatment groups exhibited comparable levels of improvement [Figure 4A]. Importantly, the number of arm entries did not differ among groups, ruling out locomotor confounds [Figure 4B]. Memory retention was further evaluated using the passive avoidance test. Aβ_1–42_-injected mice displayed significantly lower step-through latency compared to the control group, consistent with learning deficits, yet no restoration of latency time was observed in the intervention groups [Figure 4C].

Although pecan extract significantly improved spontaneous alternation in the Y-maze, no clear dose–response relationship was observed across the tested doses of 400, 800, and 1200 mg/kg. The comparable magnitude of improvement across these doses raises the possibility that the lowest tested dose of 400 mg/kg was already within a range sufficient to produce a near-maximal behavioral response under the present experimental conditions. However, because doses below 400 mg/kg were not examined, the present study cannot determine whether the response reached a true plateau or identify the minimum effective dose. Further studies incorporating lower doses, particularly those more relevant to achievable dietary exposure, are therefore warranted to better characterize the dose–response relationship.

The neuroprotective effects observed with pecan in this study are consistent with previous reports highlighting the antioxidative and anti-inflammatory activities of pecan in models of neurodegeneration. For example, a recent study showed that pecan-enriched diets improved memory-related performance and cognitive processing speed in healthy adults, indicating potential benefits for attention and memory domains [17]. Similarly, in a randomized controlled trial, a short-term dietary supplementation with pecans did not impair but supported general cognitive function in older adults, with improvements observed across multiple cognitive assessments [18]. While these studies addressed different facets of cognitive function, they collectively highlight the neuroprotective potential of pecan-derived bioactives.

To further validate the neuroprotective potential of the identified compound, an in vivo intervention study was conducted using ethyl gallate under the same experimental conditions. Cognitive function was assessed via the Y-maze test following oral administration of ethyl gallate at 10, 20, and 40 mg/kg body weight. As expected, Aβ_1–42_ injection significantly reduced spontaneous alternation behavior compared to the control group, indicating impaired working memory. However, ethyl gallate treatment at all tested doses led to improved alternation percentages, suggesting a protective effect against Aβ-induced deficits [Figure 4D]. No significant differences were observed in the number of arm entries across groups, ruling out locomotor confounds [Figure 4E]. In the passive avoidance task, Aβ_1–42_ injection resulted in markedly reduced step-through latency, indicating compromised memory retention. Treatment with ethyl gallate at 20 mg/kg partially restored latency, while the 40 mg/kg dose produced a more pronounced improvement [Figure 4F].

To evaluate potential systemic toxicity, body weight, brain weight, and serum transaminase (AST and ALT) levels were monitored, with no significant differences noted across groups, indicating that ethyl gallate was well-tolerated. Additionally, MDA levels in brain homogenates were measured to assess oxidative stress following Aβ injection. Although MDA levels tended to increase in the Aβ_1–42_ group compared to the control (0.5507 ± 0.1270 vs. 0.4137 ± 0.0356 nM/mg protein), the difference was not statistically significant. Likewise, MDA levels in ethyl gallate-treated groups (10, 20, and 40 mg/kg) were 0.4992 ± 0.1448, 0.4185 ± 0.0281, and 0.3605 ± 0.0463 nM/mg protein, respectively, with no significant differences observed compared to the Aβ group. Overall, our second animal intervention study using ethyl gallate demonstrated improvements in memory-related behavioral performance. Although brain MDA levels showed a dose-related numerical decrease following ethyl gallate administration, these differences did not reach statistical significance, and therefore the present findings are insufficient to establish a significant attenuation of brain lipid peroxidation in vivo. Notably, the behavioral profiles differed between the crude pecan extract and ethyl gallate. Pecan extract significantly improved spontaneous alternation in the Y-maze but did not significantly improve passive avoidance performance, whereas ethyl gallate showed beneficial effects in both behavioral tasks. Importantly, the ethyl gallate content of the crude pecan extract was not quantitatively determined in the present study. Therefore, the amount of ethyl gallate delivered by the pecan extract doses of 400–1200 mg/kg cannot be estimated from the current data, and these doses should not be considered equivalent to the purified ethyl gallate doses of 10–40 mg/kg. Accordingly, the two in vivo experiments should be interpreted as parallel evaluations rather than as dose-matched comparisons. The observed differences in behavioral outcomes may reflect differences in effective ethyl gallate exposure, matrix-dependent absorption or bioavailability, or the combined influence of multiple constituents present in the crude extract. Because these factors were not directly evaluated, the reasons for the differential effects in the passive avoidance test remain uncertain. Therefore, the behavioral effects of the crude pecan extract should not be attributed solely to ethyl gallate.

The behavioral findings from our second animal intervention study are generally consistent with previous reports describing the neuroprotective effects of ethyl gallate. Previous studies have also reported antioxidant properties of ethyl gallate, including free radical-scavenging activity and modulation of oxidative stress-related markers. For example, a recent study demonstrated that ethyl gallate treatment significantly lowered brain MDA levels and enhanced endogenous antioxidant enzymes, including superoxide dismutase and glutathione peroxidase, in a rodent model of neurodegeneration [19]. In contrast, although brain MDA levels showed a numerical decrease following ethyl gallate treatment in the present study, the differences were not statistically significant. Therefore, the present in vivo findings do not establish a significant antioxidant effect of ethyl gallate or demonstrate that the observed behavioral improvements were mediated by attenuation of brain lipid peroxidation.

The absence of a significant change in brain MDA further suggests that mechanisms other than attenuation of lipid peroxidation may contribute to the observed behavioral effects. Potential mechanisms may include modulation of cholinergic signaling, neuroinflammatory pathways, and Aβ-related processes. However, these pathways were not directly assessed in the present study, and their potential involvement therefore remains hypothetical and requires further experimental validation.

Aβ-induced neurotoxicity plays a pivotal role in the pathogenesis of AD, largely by promoting oxidative stress, inflammation, and neuronal degeneration. In the present study, pecan extract and ethyl gallate exhibited antioxidant and cytoprotective activities in vitro, while both treatments showed beneficial effects on memory-related behavioral performance in Aβ_1–42_-injected mice. However, brain MDA levels were not significantly reduced, precluding a definitive conclusion regarding antioxidant effects in vivo. Previous studies have reported that pecan can significantly reduce oxidative stress and enhance antioxidant enzyme activities (e.g., catalase, glutathione peroxidase, and glutathione S-transferase), ultimately lowering lipid peroxidation levels [20]. In the context of AD, bioactives (e.g., glycosaminoglycans or curcumin derivative) in natural plants may limit Aβ aggregation and deposition, reducing the formation of neurotoxic oligomers and fibrils [21] yet there are no studies specifically demonstrating that pecan can reduce Aβ-induced toxicity.

On the other hand, several studies have investigated the protective effects of ethyl gallate against Aβ toxicity, highlighting its potential in AD therapeutics. In an earlier in vitro study, ethyl gallate demonstrated potent antioxidant activity by protecting PC12 neuronal cells from hydrogen peroxide-induced cytotoxicity, suggesting its relevance to oxidative stress-associated neurodegeneration [22]. Further, following intragastric administration of a plant extract in rats, EG was detected in both plasma and brain tissues, implicating its ability to cross the blood–brain barrier and contribute to systemic antioxidant and neuroprotective effects [23]. More recently, in a diabetic rat model exhibiting cognitive impairment, intraperitoneal administration of EG (10 and 20 mg/kg) for four weeks significantly improved performance in maze-based memory tasks and reduced MDA levels in brain tissue, indicating attenuation of lipid peroxidation [19]. Furthermore, the absence of a significant reduction in brain MDA suggests that mechanisms other than attenuation of lipid peroxidation may contribute to the observed behavioral effects. Potential mechanisms may include modulation of cholinergic signaling, neuroinflammatory pathways, and Aβ-related processes. Previous studies have suggested that ethyl gallate may influence α7 nicotinic acetylcholine receptor (α7nAChR)-related pathways and facilitate Aβ clearance through autophagic mechanisms [24]. These findings provide a possible mechanistic explanation for the behavioral effects observed in the present study independently of a measurable reduction in MDA. However, cholinergic signaling, inflammatory mediators, Aβ burden, and autophagic pathways were not directly assessed in the present study; therefore, their involvement remains hypothetical and requires direct experimental validation.

While the current findings highlight ethyl gallate as a promising neuroprotective agent, several limitations warrant further investigation. An additional limitation is that food consumption was not quantitatively monitored. Because the experimental diets were provided ad libitum, individual daily intake of pecan extract or ethyl gallate could not be precisely determined, and variation in food consumption may have resulted in differences between the target and actual exposures. Future studies incorporating quantitative food-intake monitoring are therefore needed to more accurately assess individual exposure to the test materials. Future studies should aim to elucidate the specific molecular signaling pathways through which ethyl gallate modulates oxidative stress, autophagic mechanisms, and Aβ-related neurotoxicity in AD models, including the ICV administration of Aβ_1–42_ as used in our study. Long-term, chronic administration trials in transgenic AD models will be essential to determine the sustained efficacy, tolerability, and safety profile of ethyl gallate under disease-relevant conditions. Additionally, its potential synergistic effects with existing Aβ-targeted therapeutics warrant exploration to assess whether ethyl gallate can enhance or complement current treatment strategies via antioxidant or autophagy-regulating mechanisms. Nonetheless, this study presents several key strengths. First, we employed a comprehensive analytical approach to identify ethyl gallate as a bioactive constituent in pecan extract, utilizing sequential fractionation, TLC, HPLC, and GC–MS for chemical isolation and structural analyses. Second, both the crude extract and isolated ethyl gallate were evaluated in vivo, enabling comparative assessment of their neuroprotective properties against Aβ_1–42_-induced cognitive impairments. The absence of significant changes in the measured toxicity-related parameters under the experimental conditions further supports the potential of pecan and ethyl gallate as food-derived bioactives relevant to cognitive health.

In summary, this study identified ethyl gallate as a bioactive constituent of pecan extract through bioactivity-guided fractionation and chromatographic analyses. Pecan extract and ethyl gallate showed beneficial effects on memory-related behavioral performance in Aβ_1–42_-injected mice, although the magnitude and pattern of these effects differed between the two interventions. Together with the antioxidant and cytoprotective activities observed in vitro, these findings suggest that pecan-derived ethyl gallate may have potential as a food-derived bioactive compound relevant to cognitive health. Nevertheless, further studies are required to determine its bioavailability, molecular mechanisms of action, long-term safety, and efficacy in chronic or transgenic models of cognitive decline.

## 4. Conclusions

To summarize, this study demonstrated the antioxidant and cytoprotective activities of pecan (*Carya illinoinensis*) extract and identified ethyl gallate as a bioactive constituent through bioactivity-guided fractionation and chromatographic and structural analyses. In Aβ_1–42_-injected mice, dietary administration of pecan extract improved spontaneous alternation performance in the Y-maze test, while ethyl gallate improved memory-related behavioral performance in both the Y-maze and passive avoidance tests. Ethyl gallate administration did not significantly affect body or brain weight or serum AST and ALT levels under the experimental conditions. Collectively, these findings provide preclinical evidence supporting ethyl gallate as a food-derived bioactive constituent of pecan with potential relevance to cognitive health. Further studies focusing on quantitative standardization, molecular mechanisms, and long-term efficacy and safety are warranted to assess the potential application of pecan-derived ethyl gallate as a functional food ingredient.

## Figures and Tables

**Table 1:** Preliminary screening of edible and medicinal plant extracts for protective effects against oxidative stress in PC12 cells.

Scientific Name	Protective Effect (%) ^a^	Cell Viability (%) ^b^
*Tussilago farfara* L.	73.7 ± 1.7	67.1 ± 0.4
*C. ramosum Maxim*.	59.8 ± 2.4	48.4 ± 1.5
*Allium ascalonicum* L.	60.1 ± 1.6	50.7 ± 2.6
*Brassica oleracea*	31.6 ± 2.8	46 ± 0.3
*Carya illinoinensis*	78.5 ± 1.2	78.5 ± 1.3
*Ilex paraguayensis*	61.4 ± 1.1	70.1 ± 1.6
*Brassica juncea*	44.2 ± 3.9	54.9 ± 0.2
*Perilla frutescens*	35.5 ± 2.0	46.4 ± 3.1
*Prunus salicina*	33.6 ± 3.0	48.1 ± 3.2
*Ocimum basilicum*	21.3 ± 1.6	45.3 ± 3.2
*Aconitum carmichaelii Debeaux*	28.7 ± 1.5	32.4 ± 1.1
*Taraxacum officinale* Weber ex F.H. Wigg.	31.6 ± 1.2	39.1 ± 0.8
*Vigna radiata* (L.) *R. Wilczek*	49 ± 2.1	53.8 ± 1.5
*Capsicum annuum* L.	68.4 ± 1.7	67.4 ± 2.3
*Polygonatum odoratum* (Mill.) *Druce*	52.1 ± 3.2	54.3 ± 2.5
*Allium cepa* L.	43.6 ± 1.8	51.2 ± 1.7
*Citrus reticulata Blanco*	34.9 ± 1.4	47 ± 1.9

^a^ Protective effect (%) = 100 − [(DCF formation of sample group with oxidative stress − DCF formation of negative (NEG) group without oxidative stress) × 100/(DCF formation of positive (POS) group with oxidative stress − DCF formation of NEG group without oxidative stress)]. ^b^ Cell viability = formazan formation of sample group with oxidative stress × 100/formazan formation of NEG group without oxidative stress. Each extract was reconstituted in 5% dimethyl sulfoxide (*w*/*v*) to a final concentration of 1 mg/mL for screening assays. Abbreviations: NEG, untreated group; POS, H_2_O_2_-treated group. H_2_O_2_ concentrations were 100 μM for the DCF-DA assay and 200 μM for the MTT assay.

## Data Availability

The original contributions presented in the study are included in the article; further inquiries can be directed to the corresponding author.
